# The role of autophagy in hepatocellular carcinoma: friend or foe

**DOI:** 10.18632/oncotarget.17202

**Published:** 2017-04-18

**Authors:** Lian Liu, Jia-Zhi Liao, Xing-Xing He, Pei-Yuan Li

**Affiliations:** ^1^ Institute of Liver Diseases, Tongji Hospital, Tongji Medical College, Huazhong University of Science and Technology, Wuhan, China

**Keywords:** autophagy, hepatocellular carcinoma, signaling pathways, biomarkers, tumor therapy

## Abstract

Autophagy is an evolutionarily conserved lysosome-dependent catabolic process which degrades cell’s components in order to recycle substrates to exert optimally and adapt to tough circumstances. It is a critical cellular homeostatic mechanism with stress resistance, immunity, antiaging, and pro-tumor or anti-tumor effects. Among these, the role of autophagy in cancer is the most eye-catching that is not immutable but dynamic and highly complex. Basal autophagy acts as a tumor suppressor by maintaining genomic stability in normal cells. However, once a tumor is established, unbalanced autophagy will contribute to carcinoma cell survival under tumor microenvironment and in turn promote tumor growth and development. The dynamic role of autophagy can also apply on hepatocellular carcinoma (HCC). HCC is a highly malignant cancer with high morbidity and poor survival rate. Decline or overexpression of autophagic essential genes such as *ATG7*, *ATG5* or *Beclin 1* plays a key role in the occurrence and development of HCC but the exact mechanisms are still highly controversial. Signaling pathways or molecules involving in autophagy, for example PI3K/AKT/mTOR pathway, ERK/MAPK pathway, PERK pathway, p53, LncRNA PTENP1 (Long non-coding RNA PTENP1), microRNA-375 and so on, occupy an important position in the complex role of autophagy in HCC. Here, we discuss the dynamic role, the signaling pathways and the potential prognostic and therapy value of autophagy in HCC.

## INTRODUCTION

Since Christian de Duve proposed the term ‘autophagy’, which he introduced at the CIBA Foundation Symposium on Lysosomes in 1963 [1], growing enthusiasm has done much to the discovery of characteristics and functions of autophagy. Excitedly, last year’s Nobel Prize in Physiology or Medicine was about the great discovery of autophagy. This inspiring news encouraged researchers to explore more fundamental questions about autophagy. According to the different delivery modes of cargos to lysosome or vacuole, there are three primary forms of autophagy: chaperone-mediated autophagy, microautophagy and macroautophagy [2]. Among them, macroautophagy (hereafter referred to as autophagy) is the most studied, which occurs constitutively at a low level and can be further induced under stress conditions [3, 4]. More importantly, dysfunction of autophagy contributes to the pathologies of many human diseases, for instance, neurodegenerative diseases [5], fatty liver disease [6], lupus disease [7], Crohn’s disease [8], and cancers including HCC (hepatocellular carcinoma) [9].

HCC, a highly malignant cancer with a high recurrence rate and a poor prognosis, is the third leading cause of cancer death worldwide and the second leading cause of cancer death among men in less developed countries [10, 11]. Although surgical resection or transplantation helps to improve survival rates of patients, there is still no effective treatment for the advanced patients who are not eligible for surgery. It results in only a median OS (overall survival) of 6.6 months for these patients [12, 13]. Therefore, it is imperative to explore effective and safe prognostic biomarkers and therapeutic targets for the advanced HCC patients.

Autophagy probably involves in both the promotion and prevention of cancer, and its roles may be changed during tumor progression [14, 15]. It helps to carcinoma cell survival under tumor microenvironment including chemotherapies while suppresses tumor initiation in normal liver by ensuring the normal function of cells [16]. Unfortunately, the exact mechanisms are not fully understood until now. Since the dynamic and fundamental role of autophagy in HCC, this review focuses on elucidating cellular and molecular characteristics of autophagy, dynamic role and pathways of autophagy in HCC, and autophagy-related potential prognostic biomarkers and therapeutic targets for HCC.

### Characteristics and physiological roles of autophagy

#### The characteristics of autophagy

The discovery of autophagy essential gene *ATG1* has led to a recent upsurge in autophagy exploration [17], which followed by identification of dozens of related genes. Up to date, multiple laboratories have found almost 40 *ATG* genes in yeast, many of which have orthologs in higher eukaryotes [18]. The ATG protein products of *ATG* genes (hereafter, italic for the gene, non-italic for the protein) involving in autophagosome formation consist of four function units and some additional factors in mammalian: (a) ULK1 complex; (b) PI3KC III (Class III phosphatidylinositol 3-kinase complex); (c) ATG12 conjugation system; (d) LC3 (microtubule-associated protein light chain 3) conjugation system; and additional factors WIPI-2, ATG2, and ATG9. Functions and components of these units are summarized in Table 1.

**Table 1:** Essential ATG proteins involved in autophagosome formation in mammalian

Function units	Known or possible role	References
**ULK1 complex**		
ULK1	Binds with ATG13, ATG101, and FIP200	[19, 20, 163]
ATG13	Bridges the interaction of ULK1 and FIP200; is required for the localization and stability of ULK1 and stimulates the kinase activity of ULK1	[164]
ATG101	Interacts with ULK1 in an ATG13-dependent manner; regulates the stability and basal phosphorylation of ATG13 and ULK1	[24, 25, 164]
FIP200	Scaffold protein, binds with ULK1 and ATG13; is required for proper localization, stability and kinase activity of ULK1	[19, 20, 29, 164, 165]
**PI3K complex III**		
Beclin 1	BH3-only protein; interacts with Bcl-2	[28, 29, 39]
ATG14L	Enhances Vps34 lipid kinase activity, upregulates autophagy	[28, 30]
hVps15	Ser/Thr protein kinase, is required for Vps34 membrane association	[166]
Vps34	PI3K, forms Phosphatidylinositol 3-phosphate (PI3P) for autophagy	[167, 168]
**ATG12 conjugation system**		
ATG5	Forms isopeptide bond with ATG12	[31, 169]
ATG12	Ubiquitin-like protein; forms isopeptide bond with Atg5 conjugation	[31, 169]
ATG7	E1-like activating enzyme, catalyzes ATG12–ATG5 and LC3-II-PE conjugation	[31]
ATG10	E2-like activating enzyme; catalyzes ATG12–ATG5	[31]
ATG16L1	Conjugates to ATG12-ATG5 complex	[170]
**LC3 conjugation system**		
LC3	Ubiquitin-like protein, conjugates to PE, an important marker of autophagy	[34, 171]
ATG4	Cysteine protease, regulates the level of free LC3-I	[31, 34]
ATG7	E1-like activating enzyme, catalyzes ATG12–ATG5 and LC3-II-PE conjugation	[31, 34]
ATG3	E2-like conjugating enzyme analog, catalyzes LC3-II-PE conjugation	[31, 34]
**Other essential proteins**		
WIPI-2	Mammalian homolog of yeast Atg18, a PI3P-binding protein, facilitates LC3 lipidation	[35, 172]
ATG2	Probably plays an essential role at late step of autophagosome formation	[29, 36]
ATG9	The only known transmembrane protein, may contribute to membrane recruitment	[37]

**Figure 1:**
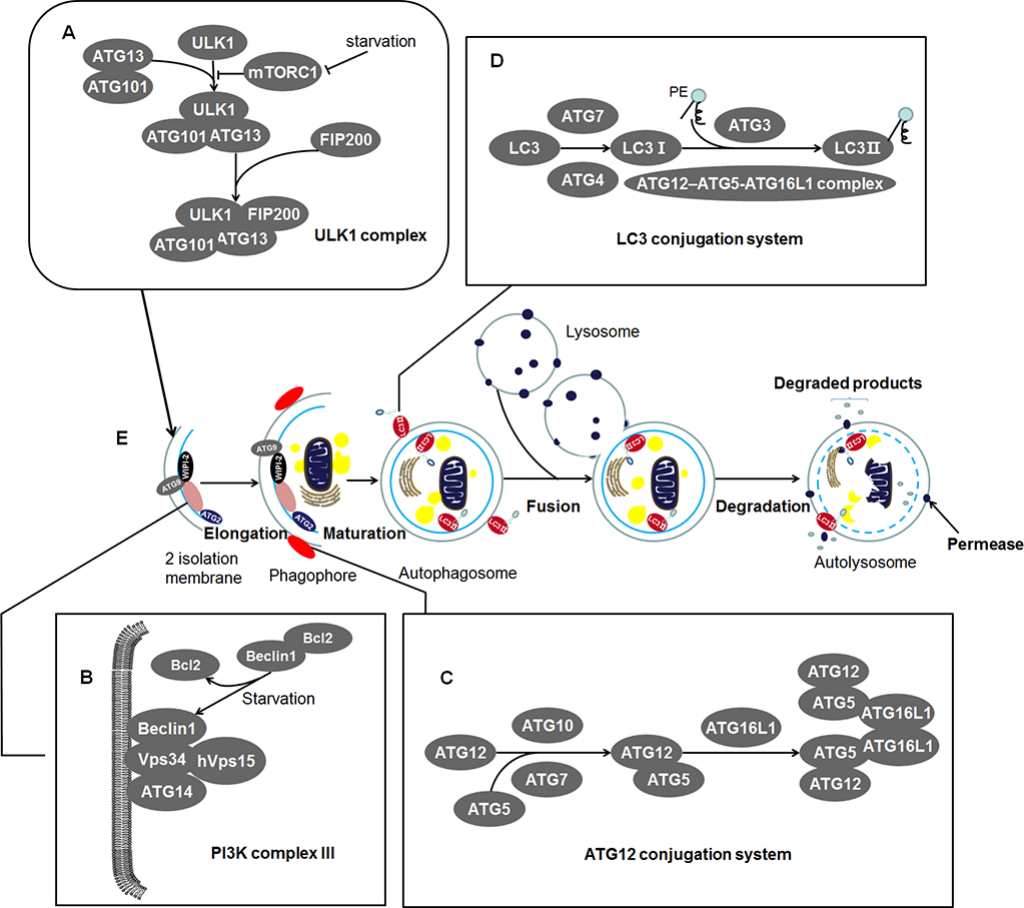
The characteristics of autophagy in mammals: Contents in the enlargement frames represent composition of the bilayer membrane (**A**) ULK1 complex: An initiator of autophagy which locates at the phagophore. Under starvation condition, the mTORC1 is blocked and the activated ULK1 and ATG13 induce the initiation of autophagy. ATG101 participates in the process in an ATG13-dependent manner and FIP200 is another critical protein for the induction of autophagy. (**B**) PI3K complex III: Recruiting the subsequent ATG proteins onto phagophore membrane. It can be negatively regulated by binding of Bcl2 and Beclin 1 while Bcl2 will release Beclin 1 during starvation. This structure locates at inner membrane of the phagophore. (**C**) ATG12 conjugation system: ATG12 is irreversibly conjugated to ATG5 in an ATG7- and ATG10-dependent manner. The ATG12-ATG5 complex binds to ATG16L1 and then forms multimerization at the phagophore. (**D**) LC3 conjugation system: LC3 is processed by ATG4 and ATG7 to generate activated LC3-I. The ATG12–ATG5-ATG16L1 complex and ATG3 involve in the conjugation of phosphatidylethanolamine (PE) to LC3-I to create LC3-II. And then LC3-II inserts into the extending phagophore membrane. LC3-II locates at two sides of the phagophore until the formation of autophagosome, which is cleaved from the outer membrane of this structure. (**E**) Morphological characteristics of autophagy: During starvation, the ULK1 complex initiates the generation of the 2 isolation membrane which extends to form phagophore. The phagophore then fully surrounds its cargos including unfolded proteins, cytoplasm, superfluous or damaged organelles. These cargos are sequestered by phagophore and fuse to form the closed autophagosome. Among the essential proteins, WIPI-2, ATG9, and ATG2 proteins locate at phagophore and exert their unsubstituted role in the formation of autophagosome. Subsequently, autophagosome merges with lysosome in mammalian cells to form an autolysosome. Eventually, the inner membrane and the enclosed cargos is degraded by protease or nuclease.

Once mTORC1 (mammalian target of rapamycin complex 1) is blocked in amino acid-deficient conditions, the most upstream unit ULK1 complex is activated, followed by nucleation and expand of phagophore (an expanding membrane sac) (See Figure 1A and 1E). The ULK1 complex is assembled by ULK1 (mammalian homolog of yeast Atg1), ATG13, FIP200 (focal adhesion kinase family interacting protein of 200 kD, also known as retinoblastoma 1-inducible coiled-coil 1, RB1CC1), and ATG101 (See Figure 1A). It initiates the formation of autophagosome and links cellular nutrient status to downstream events in autophagy [19–26]. Activated ULK1 complex leads to recruiting of PI3KC III to phagophore [27]. Autophagy-specific PI3KC III, downstream of ULK1 complex, consists of Beclin 1 (a mammalian homolog of yeast Atg6), ATG14, Vps34, and hVps15, recruiting the subsequent ATG proteins onto phagophore membrane (See Figure 1B) [28–30]. The following units are the two ubiquitylation-like modification systems—ATG12 conjugation system and the LC3 conjugation system which contribute to the elongation of the phagophore (See Figure 1C, 1D) [31–34]. In addition, proteins WIPI-2, ATG2, and ATG9 also play irreplaceably role in the membrane recruitment and the formation of autophagosome, which are described in Table 1 [29, 35–37]. These autophagic factors gather tightly at ER (endoplasmic reticulum) associated structures called omegasomes [26, 38]. The phagophore then fully surrounds its cargos including unfolded proteins, cytoplasm, superfluous or damaged organelles and fuses to form the closed autophagosome [18, 39]. Subsequently, autophagosome merges with the lysosome in mammalian cells to form an autolysosome [18]. Eventually, the inner membrane and the enclosed cargos are degraded by protease or nuclease (See Figure 1E) [39]. By this way, the degradation products of cytoplasm portions will be cycled for energy generating and substrate supplying [40]. Thus, this process can be referred as a material recycling system and energy efficient utilization mechanism under nutritional starvation or other tough conditions such as tumor microenvironment.

Regulation of ATG proteins activity plays a critical role in the balance of autophagy in mammalian cells. Increasing evidence indicates that PTMs (posttranslational modifications) of ATG proteins have significant flexibility for the balance of autophagy [41, 42]. Different PTMs play critical role in the promotion or inhibition of ATG proteins in different stages of autophagy and can trigger distinct responses in autophagy [43]. Besides, ATG proteins can be modified multiple times by different PTMs or the same PTM at different residues [43]. For example, ULK1 maintains its kinase activity through autophosphorylation at Thr180 [44], promotes its self-association and activity during autophagy by ubiquitination [45], stimulates starvation-induced autophagy by acetylation at Lys162 [46]. These complex and precise PTMs regulate activity of ATG proteins during autophagic process which will provide new therapeutic targets for the treatment of autophagy-associated diseases [43].

Collectively, autophagy is a very complex process that consists of ATG proteins and other components to assemble the required machinery [26].

### Physiological functions of autophagy

Researches on autophagy and tumor suppressing began to spring up since the relationship between autophagy and malignant transformation has been established. Decrease in autophagic capacity was observed in numerous cancers [47, 48]. For instance, lower autophagic capacity is detected in chemical carcinogens induced preneoplastic liver nodules cells and primary rat HCC cells when compared with normal cells [49, 50]. *Beclin 1* (mammalian homolog of yeast *Atg6*), an essential autophagy gene, has been found mono-allelically deleted in various cancers including liver, ovarian, breast, prostate, glioma, colon, and brain [51–57]. Besides, *Beclin 1* is not the only one gene which binds autophagy and tumor, several oncogenes or tumor suppressor genes affect autophagy-related pathways, the *PTEN* tumor suppressor genes and the *Akt, Ras* or *Myc* oncogenes, for example [58–63]. Accordingly, it is not difficult to conclude that there are numerous undiscovered genes or mechanisms exerting tumor suppressing role by contacting with autophagy.

Autophagic cell death, type-II programmed cell death, is a form of non-apoptotic cell death mechanism in which autophagy is a part of the events leading to cell death [64, 65]. Yu et al. had reported that *ATG7* and *Beclin 1*, two essential autophagy genes, were necessary for apoptotic independent cell death in mammalian cells. Furthermore, *ATG5* and *Beclin 1* could also mediate death of MEFs (murine embryonic fibroblasts) whose apoptotic cell death had been blocked [65, 66]. Both of the studies indirectly revealed that autophagy played an important role in cell death when apoptotic death was blocked.

Autophagy plays an essential role in intracellular quality controlling through the turnover of cytoplasmic components [67]. It has been reported that abnormal ubiquitinated proteins and organelles rapidly accumulated in the cytoplasm in autophagy genes *ATG5* and *ATG7* deficient liver tissue. And liver-specific *ATG7*^−/−^ mice can develop hepatomegaly and hepatic failure which indicates the remarkable position of autophagy in liver metabolism [68]. Once basal autophagy dysregulated, not only liver, but also nervous system, cardiovascular system and many other tissues can be dysfunctional [67, 69, 70]. More importantly, increasing level of autophagy accelerates the turnover of amino acid in starvation conditions. For instance, autophagy is responsible for degradation of over 30% of total proteins in liver within one day in innutritious mice [68]. And autophagy derived amino acids can also be utilized for energy providing by the TCA (tricarboxylic acid) cycle which contributes to survival of cells [71]. Briefly, autophagy keeps cellular homeostasis by intracellular quality controlling and then promotes the survival of cells.

In addition to the above functions, autophagy also plays a key role in determining life span [72]. The antiaging effect of autophagy is not only reflected in mammals but also in other organisms, such as yeast, *C. elegans*, and *Drosophila* [72, 73]. Autophagy is downregulated at the transcriptional level during normal human brain aging [74]. And dropping of BEC-1 (a Beclin 1 ortholog) could weaken the significantly extended life span of *C. elegans* [75]. In addition, CR (caloric restriction), the major antiaging measure, is the optimal physiological inducer of autophagy [76, 77]. Inhibition of autophagy will weaken the antiaging effects of CR in multiple species, such as *C. elegans* [78], *D. melanogaster* [79]*, Drosophila* [80], and mice [72, 81]. Of course, there are lots of other mechanisms involving in aging regulation. Autophagy is not necessary but, at least in some ways, also significant for life extension. Considering these reasons, it is possible that the promotion of autophagy may reduce the occurrence of time-dependent diseases and may provide a perfect introduction for aging research.

### Roles of autophagy in hepatocellular carcinoma (HCC)

The characteristics of autophagy have been extensively investigated for more than 40 years, however, the actual functions of it in HCC are still not well known. Generally, the role of autophagy in liver cancer is not unchangeable but dynamic and easily affected [82]. During dysplastic phase in hepatocytes, basal autophagy acts as a tumor suppressor by removing newly damaged mitochondria and mutated cells and thus maintaining genomic stability. However, once a tumor is established, unbalanced autophagy will contribute to HCC cell survival under various stress conditions and in turn promotes tumor growth [83]. And autophagy inhibitors exerted a tumor-suppressive effects in the HCC rat model in tumor-forming stage while had a tumor-promoting effect in dysplastic stage [83]. Hence, the role of autophagy in the occurrence and development of HCC is dependent on the context of liver cells.

### Anti-tumor role of autophagy in liver cancer

The key *ATG* genes play critical role in the activation and the occurrence of autophagy. For example, the deletion of *Beclin 1*, *ATG5*, or *ATG7* were found to associate with the tumor phenotype of HCC. In animal models the *Beclin 1*^+/−^ mutant mice developed increased frequency of spontaneous malignancies including HCC [84, 85]. Meanwhile, expression levels of *Beclin 1* were negatively related with HCC grades which in some case confirmed the positively significance of *Beclin 1* to suggest the grades of HCC [86]. In addition, *Atg5^flox/flox^* CAG-*Cre* mice, generated by crossing *Atg5^flox/flox^* mice with CAG-*Cre* transgenic mice (CAG-*Cre* transgenic mice expressing Cre recombinase under the control of the CMV enhancer and chicken β-actin promoter), was incompletely deleted and prevented the lethal phenotype of *Atg5*^−/−^. This kind of mice developed *Atg5*-deficient hepatocytes derived tumors only in liver which revealed the hepatic specific antitumor effect of autophagy [87]. Mice with liver-specific knockout of *Atg7* also developed liver tumors in some degree [87]. The results of loss of key autophagic genes suggest that basal autophagy occupies an important position in preventing the occurrence and development of liver tumors.

Furthermore, p62 (a substrate of autophagy) accumulation induced by loss of autophagy contributes to hepatic tumor formation. It has been reported that tumors in p62 transgenic animals showed more active irregular mitosis and higher expression levels of IGF2 (Insulin-like Growth Factor II). However, *Atg7*-deficient mice with p62-deficient hepatocytes showed decreasing in tumor size. Besides, conclusion from a HBV-positive HCC cohort was consistent with the above results [87, 88]. These appear that autophagy deficiency causes accumulation of p62 resulting in development of HCC. Indeed, autophagy deficiency increases the accumulation of damaged mitochondria, causes oxidative stress, and suppresses synthetic lethal deficiency in DNA repair which lead to chronic tissue damage and genome mutations in HPCs (hepatic progenitor cells) [89, 90]. The chronic damage and genome mutations of liver cells are the key factors of oncogenesis.

LncRNA PTENP1 (Long non-coding RNA PTENP1), a pseudogene of the tumor suppressor gene PTEN, induces autophagy as a pro-death response to suppress hepatocellular carcinoma [91, 92]. LncRNA PTENP1 acts as a competitive endogenous RNA and captures the miR-17, miR-19b, and miR-20a that target PTEN which inhibits the activation of PI3K/AKT signaling, thus preventing PTEN from being silenced. Therefore overexpression of LncRNA PTENP1 indirectly inhibited the PI3K/AKT pathway through PTEN overexpression and then induced pro-death autophagy resulting in death of HCC cells [92, 93]. It means that LncRNA PTENP1 induced autophagy may act as an inhibitory factor for HCC cells survival.

In addition, we investigated the role of IFN-γ which has a growth inhibitory effect on HCC and found that autophagy contributed to IFN-γ induced proliferation inhibition as well as cell death in HCC cells. In our study, IFN-γ inhibited the cell growth of Huh7 HCC cells with non-apoptotic cell death which was proved to be autophagy later. Subsequently, we confirmed that IFN-γ stimulated autophagosome formation and encouraged autophagic signals changes and autophagic flux in HCC cells. Last but not least, blocking of autophagy abolished the proliferation inhibition and cell death effects of IFN-γ on HCC. The results suggested that autophagy was essential for the proliferation inhibition effects of IFN-γ on HCC cells [94].

All these lines of evidence elucidate that autophagy mediates anti-tumor effects and participates in various signaling pathways directly or indirectly to prevent the occurrence and progression of hepatocarcinoma.

### Pro-tumor role of autophagy in liver cancer

However, the intracellular quality control and the amino acid cycle functions of autophagy may become an accomplice of cancer cell survival in starvation, hypoxia, metabolic stress and other stress conditions [16]. In other words, cells can survive this self-digestion to get an alternative energy source under tumor microenvironment. A 156 HCC patients study reported that the expression levels of LC3-II (a key autophagic marker) in HCC were associated with vascular invasion (*P* = 0.016), lymph node metastasis (*P* = 0.042), and TNM stage (*P* = 0.037). Moreover, the overexpression of LC3-II predicted an inferior 5-year OS (overall survival) rate (*P* = 0.026), which suggested that the expression levels of autophagy were positively related with the development and a poor prognosis of HCC [95]. Indeed, increased autophagy has been detected in advanced liver cancer, and is closely related to malignant transformation and low survival rate in HCC patients [95–97].

Our team investigated the role of miR-375 which is one of the most significantly downregulated miRNAs in HCC and found that it inhibited autophagy by reducing the expression of Atg7 and then decreased viability of HCC cells under hypoxic conditions in culture and in mice. MiR-375 suppressed the conversion of LC3-I to LC3-II under hypoxic conditions which thereby blocked autophagic flux, inhibited mitochondrial autophagy of HCC cells, reduced the elimination of damaged mitochondria, increased releasing of mitochondrial apoptotic proteins, and then impaired viability of HCC cells [98, 99]. The results indicate that autophagy promotes the survival of HCC cells under hypoxia in established HCC cells.

Autophagy encourages the development of liver cancer via inhibiting the expression of tumor suppressors or contributing to the chemoresistance of HCC cells. It had been argued that the above liver-specific *Atg5* deficient mice developed only hepatic adenoma but not hepatic cancer was induced by the expression of tumor suppressors such as p53. Besides, Tian et al. found that impaired autophagy suppressed the development of HCC through inducing of tumor suppressors such as p53, p16, p21, and p27 [100]. That is to say, HCC induced autophagy may block the anti-tumor effects of various tumor suppressors and blocking of autophagy may be an ideal target for the therapy of established HCC. Moreover, autophagy contributes to the chemoresistance of HCC cells. It has been reported that hypoxia induced autophagy decreased apoptotic potential of hepatocarcinoma cells resulting in chemoresistance. And inhibition of autophagy made HCC cells sensitive to chemotherapeutic agents. Sorafenib, the only FDA (Food and Drug Administration) approved HCC systemic therapy for instance, can stimulate the expression of multiple autophagy markers in HCC cells *in vitro* [101, 102]. Some works have established that sorafenib induced autophagy acts as a chemoresistance mechanism in HCC. When the autophagic key genes *Beclin 1* or *Atg5* have been suppressed, sorafenib will kill more cancerous cells and its antiproliferative ability improves. These suggest that autophagy inhibitors may play a synergistic anti-tumor effect with chemotherapy [101]. In addition, autophagy also assists activated Ras proteins to maintain tumorigenesis [103–105]. In short, autophagy plays its full part through many direct or indirect ways ultimately to promote the growth of HCC cells.

Taken together, all these lines of evidence suggest that autophagy can act as an accomplice of survival, malignant progression and distant metastasis of hepatocellular carcinoma cells in tumor-forming stage. The pro-tumor role of autophagy in hepatocarcinoma is dependent on the stages of tumor development. And the inhibition of autophagy may be an effective anti-tumor mechanism in established liver cancer cells.

### Signaling pathways of autophagy in HCC

#### PI3K/AKT/mTOR pathway

**Figure 2:**
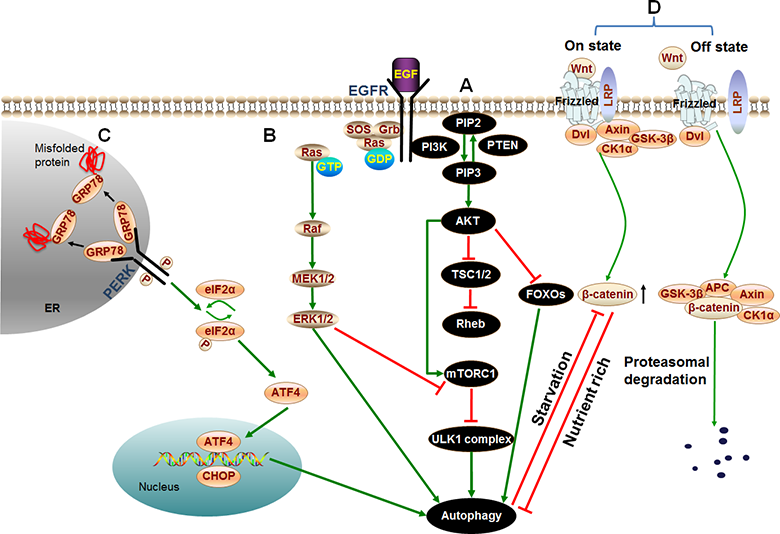
Signaling cascades of autophagy in HCC (**A**) PI3K/AKT/mTOR pathway: Binding of growth factors to the corresponding receptors such as EGFR triggers PI3K. Then activated PI3K catalyzes the production of PIP3 which phosphorylates and activates the AKT serine/threonine kinase. Subsequently, the phosphorylated AKT activates mTORC1 resulting in repression of autophagy. Besides, activated AKT causes inactivation of TSC1/TSC2 that eventually leads to the activation of Rheb. Rheb subsequently activates mTORC1 which results in the same effect on autophagy. In addition to activating mTORC1 directly or indirectly, active AKT can also directly regulate transcription factors FOXOs resulting in inhibition of autophagy. (**B**) ERK/MAPK pathway: Extracellular molecules EGF, IGF or the like, bind with cell surface receptors EGFR or IGFR which then stimulate Ras. Therefore, Ras switches from inactive (GDP-bound) to the active (GTP-bound) form. Activated Ras binds to and recruits Raf kinases to the cell membrane for Raf dimerization and activation. Subsequently, activated Raf phosphorylates and activates MEK; MEK in turn phosphorylates and activates ERK/MAPK. Last, phosphorylated ERK directly activates autophagy or phosphorylates a variety of substrates in the membranes, cytoskeletal compartments, cytosol, and nucleus which can trigger autophagy by inhibiting mTORC1. (**C**) PERK pathway: Under conditions of accumulation of misfolded or unfolded proteins, GRP78 binds to these proteins, permitting the release of PERK, leading to activation of PERK. Activated PERK then phosphorylates eIF2α. PERK-eIF2α activation paradoxically increases the translation of ATF4, ATF4 activates transcription factor CHOP homologous protein. Last, ATF4 and CHOP cooperatively induce autophagy. (**D**) Wnt/β-catenin pathway: In the absence of Wnt signal (Off state), β-catenin is phosphorylated by APC/Axin/GSK-3β complex, resulting in the ubiquitination and proteasomal degradation of β-catenin. However, in the presence of Wnt ligand (On state), GSK-3β is displaced from the APC/Axin/GSK-3β complex which prevents β-catenin from being phosphorylated and subsequent proteasomal degradation. Thus, the concentration of β-catenin is increased in cytoplasm. A growing number of β-catenin limits autophagy in nutrient rich condition. While β-catenin itself is specifically targeted for degradation by autophagy in starvation stress.

PI3K/AKT/mTOR pathway is a well-known signaling pathway which regulates cell growth, survival, metabolism and apoptosis in physiological conditions and takes great importance for the development and survival of multiple solid tumors including HCC [106–112]. AKT, the key factor of the signaling pathway, mediates the inhibition of autophagy through a variety of ways (See Figure 2A). Contrarily, PTEN, the phosphatase and tensin homolog, reduces the level of PIP3 and initiates the formation of autophagosome [113]. PTEN has a strong phosphatase activity for dephosphorylating PIP3 to become PIP2 which contributes to the inactivation of AKT [114]. PTEN inhibits PI3K/AKT signaling and is frequently reduced or mutated in human cancers [115, 116]. These reveal that PI3K/AKT signaling may be positively related with the proliferation and migration of HCC. Actually, a variety of studies had indicated that repression of the PI3K/AKT/mTOR signaling pathway restricted the growth of HCC cells. A recent report suggested that knockdown of PI3K/AKT/mTOR suppressed the proliferation and migration of HCC cells [118], which demonstrated that inhibition of PI3K/AKT could be an effective molecular target for HCC therapy. Furthermore, arenobufagin (a bufadienolide from toad venom) suppressed the growth of HCC cells by inducing the initiation of autophagy in human hepatocellular carcinoma cells through inhibition of PI3K/AKT/mTOR pathway. [119]. It suggests that blocking of PI3K/AKT/mTOR signaling can induce the occurrence of autophagy and contribute to the suppression of HCC.

All these lines of evidence suggest that suppression of PI3K/AKT/mTOR signaling pathway induces the initiation of autophagy, and then inhibits the proliferation and migration of HCC cells.

### ERK/MAPK pathway

ERK/MAPK pathway, also known as RAF/MEK/ERK pathway, is an evolutionarily conserved signaling pathway that responds to signals from cell surface receptors to promote cell growth, proliferation, survival, and differentiation [120]. More significantly, the frequent mutations in members of this pathway are believed to contribute to tumorigenesis, tumor progression and metastasis in many solid tumors including HCC [121–124]. A recent clinical survey showed that BRAF (mutated RAF paralogue) mutation was associated with a poor median RFS (recurrence free survival rate), higher recurrence rate and mortality after hepatic cancer resection. It suggested that BRAF mutation could be an effective prognostic marker after surgical treatment of liver cancer [125]. Furthermore, a 46 HCC cases study showed that activation of MEK1/2, overexpression of ERK1/2, and hyperphosphorylation of ERK1/2 were respectively detected in 100%, 91% and 69% of HCC patients [126]. Accordingly, abnormal expression of members of RAF/MEK/ERK pathway contributes to the development of HCC.

Moreover, ERK can trigger autophagy by inhibiting mTORC1 (See Figure 2B) [127]. Activated Ras can induce high levels of basal autophagy in human cancer cell lines during nutrient rich state and requires autophagy to maintain its effect of tumorigenesis (See Figure 2B) [103, 128]. Briefly, ERK/MAPK pathway promotes the development of tumors through various ways including the induction of autophagy. However, whether the HCC promotion role is mediated by autophagy or not is rarely reported. Inhibition of members of ERK/MAPK pathway could block the progression of HCC cells. Indeed, researchers have been devoted much energy to the research of pathway inhibitors, some of which now have been found or even been approved for treatment of tumors [102, 129].

### PERK pathway

PERK (PRKR-like ER kinase) pathway is one of the signal branches of UPR (unfolded protein response). UPR is triggered by accumulation of excessive unfolded proteins when the unfolded proteins exceed the protein synthesis and folding capacity of ER (endoplasmic reticulum) [130]. It has been reported that autophagy was the downstream of the PERK signal axis and eventually led to survival of tumor cells (See Figure 2C) [131]. However, the PERK signal axis can also mediate the apoptosis of HCC cells through autophagy. The insufficient correction of misfolded proteins could induce the apoptosis of tumor cells [132]. Due to the dynamic functions of PERK induced autophagy, balance of the PERK signal in HCC cells could sensitize cancer cells to tough environment [132, 133].

### Wnt/β-catenin signaling pathway

Increasing evidences demonstrated that the Wnt/β-catenin pathway played a major role in hepatic oncogenesis [134, 135]. In the presence of Wnt ligand, the pathway is on state and β-catenin (a transcription factor as a key component of the Wnt signaling pathway) is increasing (See Figure 2D). Then β-catenin enters the nucleus and coordinately up-regulates the expression of target genes involved in cell proliferation, migration, invasion, cell cycle progression, and metastasis, which facilitate the development of HCC [135–137].

Besides, Wnt/β-catenin signaling can negatively regulate autophagy. β-catenin acts as a transcriptional co-repressor of p62 to limit autophagy in nutrient rich condition. Interestingly, β-catenin is itself directly interacting with LC3 and hence is specifically targeted for degradation by autophagy in starvation stress (See Figure 2D). These results suggest that autophagy and Wnt/β-catenin signaling crosstalk mechanism occupies an important role in the regulation of cell proliferation [138]. However, the relationship among autophagy, Wnt/β-catenin pathway, and HCC has not been reported until now. Considering the abnormal expression of β-catenin in HCC and the relationship between autophagy and β-catenin [139–141], it could be a new target for HCC research to investigate the relationship among β-catenin, the dynamic role of autophagy, and the development of HCC.

### Other pathways

In addition to the above pathways, there are many other signaling pathways which are both associated with autophagy and hepatocarcinoma. For example, HGF/c-MET signaling suppresses autophagy via interaction with PI3K/AKT pathway while overexpression of *c-MET* was observed in HCC samples [142, 143]. By promotion or inhibition of autophagy, these pathways affect the development of HCC [144, 145].

### Potential prognostic biomarkers and therapy value of autophagy in HCC

#### Prognostic biomarkers of autophagy in HCC

HCC is the third leading cause of cancer death worldwide with a strong ability of invasion and metastasis [10]. Although surgical resection or transplantation helps to improve survival rates of patients, there is still a high recurrence rate and a poor long-term survival in HCC patients [146]. It is critical to predict individual recurrence risk and prognosis of HCC. Unfortunately, current biomarkers such as AFP (alpha-fetoprotein) cannot meet the specificity and sensitivity of prognostic prediction [147]. Therefore, more sensitive and more specific prognostic biomarkers are required for effective early diagnosis of HCC recurrence.

LC3-II, a widely used autophagic biomarker, was revealed to play a significant role for the development of cancer and associated with the poor survival of cancer patients [96, 148–150]. Wu et al. reported that LC3-II was overexpressed in tumor region when compared with normal adjacent tissues and the expression levels of LC3-II were positively related with vascular invasion and lymph node metastasis of HCC patients. Besides, they found abnormal expression of LC3-II predicted a lower OS for early HCC. All these lines of evidence revealed that LC3-II was a potential biomarker of OS for HCC [95].

The expression level of Beclin 1 may be a valuable OS marker for HCC. A study on 103 primary HCC patients showed that the levels of Beclin 1 were significantly lower in HCC tissues than in adjacent tissues (72.8 vs. 89.5%, *P* = 0.015). Meanwhile, Cox regression analysis revealed that Beclin 1 expression was an independent indicator for the OS of HCC patients (*P* < 0.05) [151]. It is not hard to understand that the expression level of Beclin 1 may be a valuable prognostic marker of liver cancer and loss or lower expression of Beclin 1 may suggest an inferior prognosis of HCC.

However, it is difficult to understand autophagy-associated proteins LC3-II and Beclin 1 can be negatively related. This is, perhaps, because the two proteins are involved in different autophagic steps and interrelated with each other under certain conditions. Besides, the use of autophagy-associated markers such as LC3-II must be complemented by assays to estimate overall autophagic flow, level of LC3-II alone cannot demonstrate the overall level of autophagy [148]. Likewise, low expression level of Beclin 1 alone does not indicate a decrease in autophagy. Autophagic flux could be assessed by following turnover of LC3-II in the absence and presence of autophagy inhibitors, and by examining the autophagy-dependent degradation of appropriate substrates, such as p62 [148]. However, as a ubiquitous cellular event, autophagy is related to various of biological and physiological process. Therefore, autophagy or autophagic biomarkers such as LC3-II would be combined with other biomarkers such as alpha-fetoprotein (AFP) or with imaging diagnosis to improve the specificity of HCC prognostic prediction.

### Potential therapy value of autophagy in HCC

The pro-tumor role of autophagy raises the possibility that autophagy inhibition may be an advantage for cancer therapy [16, 104]. Actually, efficacy of autophagy inhibitors has begun to be assessed in animal models and clinic, and the results are encouraging. Given that autophagy is an accomplice of cancer cell survival in stress conditions, autophagy inhibitors may enhance the sensitivity of cancer cells to hypoxia, metabolic stress and other tough circumstances. The tumor suppression activity of autophagy inhibitor HCQ (hydroxychloroquine), which suppresses autophagy by inhibiting the function of lysosome, has been actively assessed in mouse models [152]. The study suggested that inhibition of autophagy could enhance the death-promoting activity of tumor-suppression pathway [15]. In addition, inhibitors of autophagy such as 3-MA (3-methyladenine) which blocks the fusion between autophagosome and lysosome can enhance pro-apoptotic effects of meloxicam in HCC cells. Meloxicam is a selective COX-2 (cyclooxygenase 2) inhibitor which has anti-tumor effects on various tumors [130, 153]. And multiple ATG proteins posttranslational modifications (PTMs) can block the process of autophagy which provide new therapeutic targets for the treatment of HCC [9, 43]. Thus, inhibition of autophagy promotes the death of HCC cells but also has significant synergistic antitumor effects with antineoplastic agents.

Nevertheless, the physiological functions of autophagy are critical for some normal cells and tissues. A serious question is whether systemic autophagy defect will be sufficiently targeting to impair cancer growth while preventing normal tissues from the detrimental effects. We need to establish whether tumors are more susceptible to autophagy deletion than normal tissues, therefore providing a therapeutic window for patients who would most benefit from this treatment [16, 154].

Therefore, inhibitors of members of some autophagy-associated pathways may exert antitumor effects on HCC therapy. Indeed, inhibitors of several autophagy-associated pathways have been found to be potential targets for HCC intervention. Among the known inhibitors, sorafenib is the earliest approved molecular targeting therapeutic drug for HCC patients with liver resection or at advanced stage. It inhibits components of the RAF/MEK/ERK signaling pathway, blocks tumor growth and the pro-angiogenic factor receptors, thus inhibiting neoangiogenesis [155]. In the pivotal sorafenib phase III SHARP (Sorafenib HCC Assessment Randomized Protocol) trial, a double-blind RCT (randomized controlled trial) with a primary end-point of OS, sorafenib significantly prolonged the OS of patients with advanced HCC from 7.9 to 10.7 months (hazard ratio [HR], 0.69; 95% confidence interval [CI], 0.55–0.87; *P* = 0.001). A parallel phase III RCT measured in the Asia-Pacific region, sorafenib also showed longer median OS (6.5 *vs*. 4.2 months) in patients with advanced HCC (HR, 0.68; 95% CI, 0.50–0.93; *P* = 0.014). Although side effects were observed in both trials, sorafenib was the first and the only targeted therapy to demonstrate an OS benefit in patients with advanced HCC and was approved for clinical use in several countries [102, 129]. However, sorafenib can also induce autophagy which promotes cell survival in hepatocellular carcinoma either *in vivo* or *in vitro* by an ERK/MAPK pathway independent way [156]. Besides, a study confirmed this conclusion by analyzing the potential link between sorafenib and autophagy in patients [157]. Sorafenib could kill more HCC cells with improving antiproliferative ability when autophagy was repressed by chloroquine (CQ) or bafilomycin A1 or by a siRNA (small interfering RNA) against Beclin 1 or ATG5 [158]. Thus, autophagy contributes to the appearance of resistant cells and inhibition of autophagy could help to improve the efficiency of sorafenib. But some studies showed that sorafenib induced autophagy promoted programmed cellular death in HCC cells both *in vitro* and *in vivo* models [159]. The different results suggest that normalization of autophagy may be one of the key mechanisms to avoid cellular resistance to sorafenib or other antineoplastic agents [160, 161]. In addition to sorafenib, many other signaling pathway inhibitors such as lenvatinib and tivantinib have been in clinical trial stage or have been approved in clinical use. These inhibitors block the autophagy-associated pathways including the ERK/MAPK cascade, the PKC (protein kinase C) pathway, and the PI3K/AKT pathway [162].

Collectively, balance or inhibition of autophagy or autophagy-associated pathways could enhance the anticancer efficiency of both native tumor-suppressor mechanisms and chemotherapy to restrain tumor growth and progression.

### Conclusion and future directions

In conclusion, autophagy plays an anti-tumor role in normal liver cells by maintaining cell homeostasis, but once the tumor is formed, it promotes the survival of HCC cells under tumor microenvironment. Several vital pathways including PI3K/AKT, RAF/MEK/MAPK, PERK, and Wnt/β-catenin signaling pathways are involved in the autophagy procedure and the regulation of HCC initiation and progression. Many core molecules mentioned in these signaling pathways can be used as prognostic biomarkers or therapeutic targets for HCC. However, what we have known about autophagy in HCC so far is just the tip of the iceberg. A better understanding of the mechanisms by which autophagy exerts different functions at different stages of HCC is important and urgently needed. As autophagy is a ubiquitous cellular event, exploration on tumor-specific autophagy loss and effective pharmacological agents for blocking of autophagy in cancer cells may be a great hotspot and breakthrough point for reducing HCC risk and improving HCC therapeutic efficacy.

## Abbreviations

HCC: Hepatocellular carcinoma
PI3K: Phosphatidylinositol 3-kinase
AKT: Protein kinase B
mTORC1: Mammalian target of rapamycin complex 1
ERK: Extracellular signal-regulated kinase
MAPK: Mitogen-activated protein kinase
PERK: PKR-like endoplasmic reticulum kinase
LncRNA PTENP1: Long non-coding RNA PTENP1
OS: Overall survival
ULK1: Mammalian homolog of yeast Atg1
PI3KC III: Class III phosphatidylinositol 3-kinase complex
LC3: Microtubule-associated protein light chain 3
WIPI-2: WD-repeat protein interacting with phosphoinositides 2
FIP200: Focal adhesion kinase family interacting protein of 200 kD
RB1CC1: Retinoblastoma 1-inducible coiled-coil 1
Vps34: Phosphatidylinositol 3-kinase
hVps15: Ser/Thr protein kinase
ER: Endoplasmic reticulum
PTMs: Posttranslational modifications
MEFs: Murine embryonic fibroblasts
TCA: Tricarboxylic acid
BEC-1: Beclin 1 ortholog
CR: Caloric restriction
CMV: Cytomegalovirus
IGF2: Insulin-like Growth Factor II
HBV: Hepatitis B virus
HPCs: Hepatic progenitor cells
IFN-γ: Interferon gamma
FDA: Food and Drug Administration
PIP3: Phosphatidylinositol 3,4,5-trisphosphate
MEK: MAPK kinase
RFS: Recurrence free survival rate
UPR: Unfolded protein response
HGF: Hepatocyte growth factor
AFP: Alpha-fetoprotein
HCQ: Hydroxychloroquine
3-MA: 3-methyladenine
COX-2: Cyclooxygenase 2
SHARP: Sorafenib HCC Assessment Randomized Protocol
RCT: Randomized controlled trial
CQ: Chloroquine
siRNA: Small interfering RNA
PKC: Protein kinase C
IGFR: Insulin-like Growth Factor receptor
EGF: Epidermal growth factor
EGFR: EGF receptor
PIP2: Phosphatidylinositol-4,5-bisphosphate
GRP78: Glucose regulated protein 78 kDa
eIF2α: Eukaryotic translation initiation factor 2α
ATF4: Activating transcription factor 4
CHOP: C/EBP homologous protein
SOS: Son of sevenless
TSC1/2: Target of rapamycin complex 1/2
FOXOs: Forkhead box protein O
Rheb: Ras homolog enriched in brain
LRP: Lipoprotein receptor-related protein
Dvl: Dishevelled
GSK-3β: Glycogen synthase kinase 3β
CK1α: Casein kinase 1α
APC: Adenomatous polyposis coli
Bcl2: B cell leukemia 2
